# Pseudo-transistors for emerging neuromorphic electronics

**DOI:** 10.1080/14686996.2023.2180286

**Published:** 2023-03-20

**Authors:** Jingwei Fu, Jie Wang, Xiang He, Jianyu Ming, Le Wang, Yiru Wang, He Shao, Chaoyue Zheng, Linghai Xie, Haifeng Ling

**Affiliations:** a State Key Laboratory of Organic Electronics and Information Displays & Institute of Advanced Materials (IAM), Nanjing University of Posts & Telecommunications, Nanjing, China; b Yangtze Delta Region Institute (Huzhou), University of Electronic Science and Technology of China, Huzhou, China

**Keywords:** Neuromorphic computing, artificial synapse, pseudo-transistor, non-volatile memory, multi-terminal

## Abstract

Artificial synaptic devices are the cornerstone of neuromorphic electronics. The development of new artificial synaptic devices and the simulation of biological synaptic computational functions are important tasks in the field of neuromorphic electronics. Although two-terminal memristors and three-terminal synaptic transistors have exhibited significant capabilities in the artificial synapse, more stable devices and simpler integration are needed in practical applications. Combining the configuration advantages of memristors and transistors, a novel pseudo-transistor is proposed. Here, recent advances in the development of pseudo-transistor-based neuromorphic electronics in recent years are reviewed. The working mechanisms, device structures and materials of three typical pseudo-transistors, including tunneling random access memory (TRAM), memflash and memtransistor, are comprehensively discussed. Finally, the future development and challenges in this field are emphasized.

## Graphical abstract

**Figure:**
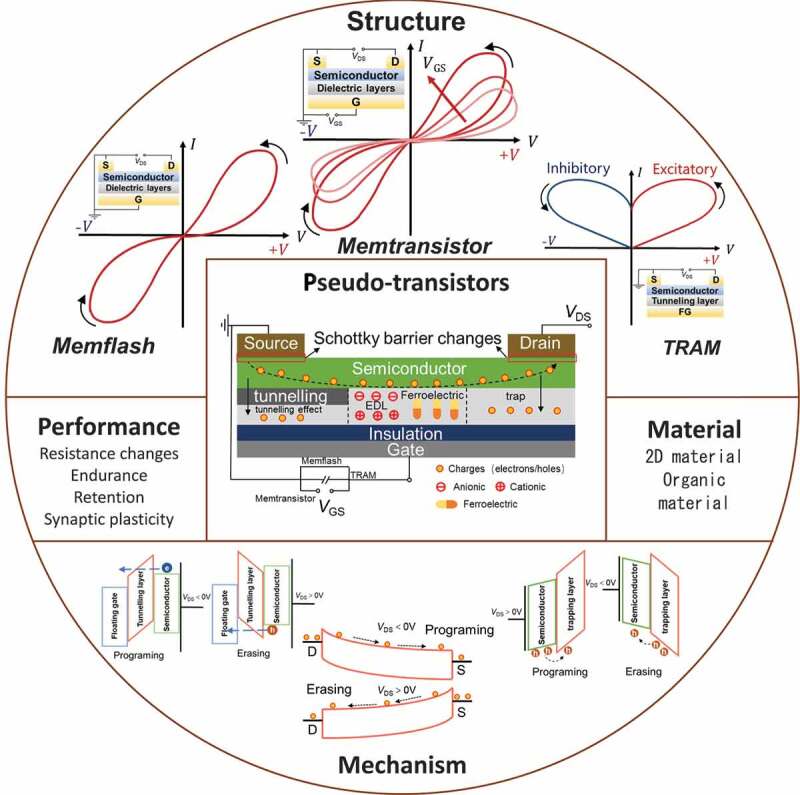

## 1. Introduction

Neuromorphic engineering, also known as neuromorphic computing, is a concept developed by Carver Mead in the late 1980s [1]. This describes the use of electronic analogue circuits to mimic neurobiological architectures present in the nervous system. As the hardware realization of artificial intelligence, neuromorphic electronics are attracting increased attention [2–5]. Neuromorphic electronic devices are the building blocks which perform learning and computing processes by mimicking the functions of biological neurons and synapses. The human brain consists of approximately 10^11^ neurons and 10^15^ synapses, and the energy consumption (~20 W) is even far less than supercomputers (~10^7^ W) [6–9]. So far, large-scale neuromorphic chip has been successfully developed with complementary metal-oxide-semiconductor (CMOS) technology [10,11], such as the Loihi announced by Intel Corporation in 2017 [12]. However, silicon neuron requires a large number of MOS transistors [13]. Strategies for building future large-scale neuromorphic circuits rely on the exploration of memory component at the single-device level, such as artificial synapses. Emerging electronic devices with tunable memory property are needed.

**Figure 1.**
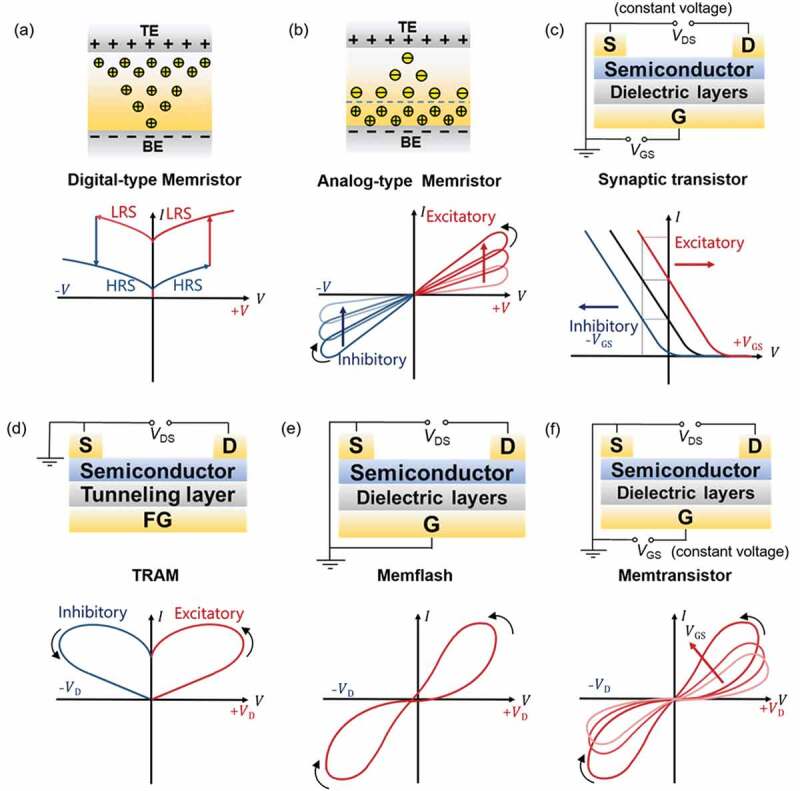
Current-voltage (*I-V*) characteristics of various synaptic devices, device structure and their wiring mode: (a) Digital-type memristor and its typical *I-V* characteristics. (b) Analog-type memristor and its *I-V* characteristics. (c) Synaptic transistor memory and its typical *I-V* characteristics. (d) TRAM and its typical *I-V* characteristics. (e) Memflash and its typical *I-V* characteristics. (f) Memtransistor and its typical *I-V* characteristics.

Currently, neuromorphic electronic devices based on two-terminal memristors and three-terminal transistors have been extensively studied [14–18]. Memristor works on the principle of electric field-driven resistance transitions, mainly including resistive random access memory (RRAM) [19,20] and phase-change memory (PCM) [21]. Figure 1(a) shows the *I-V* characteristic curve of the digital-type memristor, which realizes the data storage function by switching between a high resistance state (HRS, or off state ‘0’) and a low resistance state (LRS, or on state ‘1’) [22,23]. Figure 1(b) illustrates the analog-type memristor based on interfacial barrier modulation to realize gradually adjustable conductance states. Strukov *et al*. first proved Leo Chua’s memristor theory using Pt/TiO_x_/Pt resistive switching device [24]. Due to the redox that occurs under an external bias voltage, the device resistance varies with the history of the applied voltage or current. Sinder *et al*. reported the implementation of spike-timing-dependent plasticity (STDP) in memristive nanodevices [25]. Lu *et al*. correlated the synaptic functions with the conductance states of nanoscale silicon-based memristor [26]. Fabien *et al*. reported a crossbar circuit based on a TiO_2_ memristor and trained it by in situ and non-in situ methods [27]. Kuzum *et al*. firstly reported a PCM-based artificial synapse [28]. The conductance value can be changed by controlling the crystallization state [29]. Conductance value of different memory state can be regards as synaptic weight, which will gradually increase or decrease under consecutive voltage pulses to simulate the connection strength between neurons [30,31]. Vector-matrix multiplication can be performed directly utilizing Ohm’s law and Kirchhoff’s law in a memristor crossbar array [32,33]. For the three-terminal transistors, with the signal applied to the gate, the positive/negative shift of the transfer curves, and thus the tunable conductance states can be achieved (Figure 1(c)). Additionally, the control of the programming (gate) and reading (drain) is separated in this three-terminal configuration, which enables the flexible regulation of device conductance [34,35]. Three-terminal transistors synaptic have the advantages of good stability and reconfigurability. The channel conductance value is regarded as the synaptic weight to store information for neuromorphic computing [36–38].

In terms of neuromorphic computing, the above two types of memory devices have shown great potential for applications in pattern recognition [32,39–41], associative learning [42,43], biomimetics [44–47] and neuromorphic circuit [48–53]. As one of the most promising memory devices, memristor has faster speed and lower energy consumption, and the size of the memristor can be reduced to sub-nanometer scale [54]. Moreover, large-scale memristor array could be easily integrated in a crossbar architecture to perform vector-matrix multiplication for neuromorphic computing. However, the random growth of the conductive filament in redox memristors can reduce the reliability and reproducibility performances [55–57]. Phase-change memories suffer from resistive drift, a physical phenomenon caused by the rearrangement of atoms in phase-change materials within the amorphous phase, and thus limit the number of levels that can be reliably programmed and read back [58]. Besides, to reduce crosstalk problem of the memristor crossbar array, it is necessary to insert access devices at each node like a transistor with a high on-off ratio (i.e. 1T-1R) [8,59–61]. In this context, the concept of pseudo-transistors is proposed. The memristor characteristics can be implemented by changing the wiring mode of the conventional transistor and retaining the characteristics of the transistor itself. As a hybrid integration of memristors and transistors, a pseudo-transistor integrates the electrical properties of memristor-based 1T-1R structures into a single compact device [62]. Pseudo-transistors show remarkable advantages reconfigurable or logic neuromorphic circuits because of their combination of multi-level continuous non-volatile memory properties.

In terms of the wiring mode, the pseudo-transistors can be categorized into tunneling random access memory (TRAM) [63–65], memflash [66–68] and memtransistor [69–71]. TRAM can be regarded as a gate-free tunneling transistor, It can have extremely short channel length to achieve high memory integration [65]. Figure 1(d) illustrates the typical electrical curve of TRAM. Memflash simplifies the three-terminal structure of floating-gated transistor into two-terminal structure [67]. As can be noted from the characteristic curve in Figure 1(e). Different from the above two kinds of pseudo-transistors, memtransistor can be viewed as a gate-tunable memristor, in which the gate acts as a constant voltage terminal to modulate the hysteresis caused by source-drain terminals (Figure 1(f)) [72,73]. This provides structural advantages for achieving more complex synaptic plasticity, for example, heterosynaptic plasticity and learning-on-demand rules [74,75].

**Figure 2.**
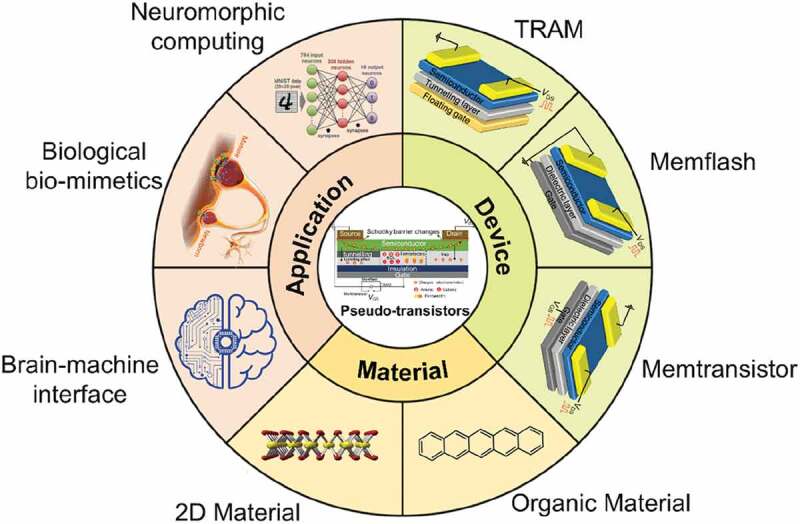
Summary of materials, device structures, and application fields of pseudo-transistors. Materials include 2D materials and organic materials. The device structures include memflash, TRAM and memtransistor. The applications of pseudo-transistors fall into three main categories: the first category is for the biomimetics of synapses, and the second category is for neuromorphic computing and the third is brain-computer interfaces. Reproduced with permission [76] Copyright 2021 WILEY-VCH, and [77] Copyright 2020 WILEY-VCH.

At present, pseudo-transistors have been studied extensively as neuromorphic electronic devices (Figure 2). In this review, we start with an introduction to biological synapses. Secondly, the materials used to prepare pseudo-transistors are described in detail. Then, the device structure, working mechanism and state-of-art research progress of pseudo-transistors are discussed. Finally, current problems in pseudo-transistor are analyzed and discussed for future research.

## 2. Biological synapse

**Figure 3.**
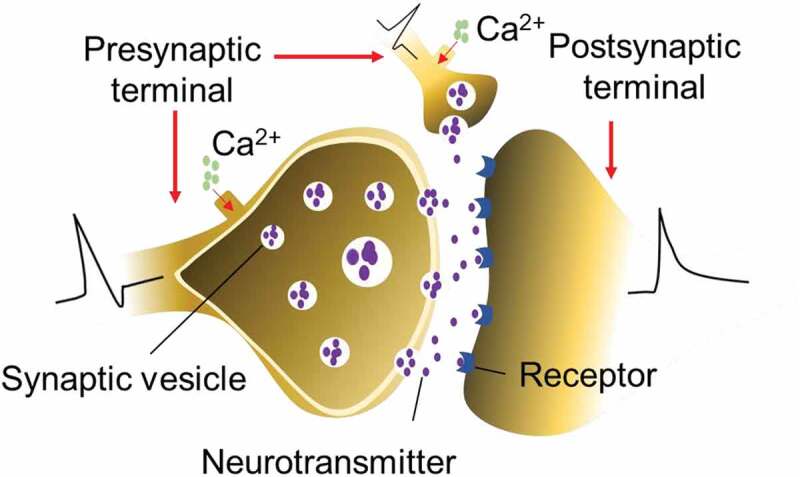
The structure of biological synapses, the input of presynaptic signals and the output of postsynaptic neuronal electrical signals.

As the junction between pre- and postsynaptic neurons (Figure 3), synapses are the basic units of neural networks and undertake the key tasks of material interaction and information transfer between neurons [78,79]. Synapses are mainly divided into two categories: chemical synapses and electrical synapses [80]. Electrical synapses transmit information with the help of electrical signals. Chemical synapses transfer information via neurotransmitter, which are found primarily in the human body. The transmission and handling of information is a complex process: firstly, action potentials control the opening of Ca^2+^ channels at presynaptic neuron, releasing excitatory or inhibitory neurotransmitters in the synaptic cleft. At the postsynaptic neuron, the neurotransmitters bind to a specific protein receptor. This receptor converts the chemical signal back into an electrical signal. In this way, the neurotransmitters can initiate an electrical response that either excite or inhibit the postsynaptic neuron.

The connection strength between neurons is called synaptic weight, and synaptic plasticity is the tunability of synaptic weight. Synaptic plasticity can be categorized into homogeneous and heterogeneous synaptic plasticities. Homologous synaptic plasticity occurs at synapses that are directly involved in cell activation during induction. This synaptic plasticity is also known as input specificity [81]. Heterogeneous synaptic plasticity complements homogeneous synaptic plasticity by regulating the activity of postsynaptic neurons through neuromodulatory transmitter to avoid hyperexcitation or hyperinhibition [81,82]. Besides, synaptic plasticity can also be divided into short-term (STP) and long-term plasticities (LTP) [83]. STP is a temporary change in synaptic weights, lasting from a few milliseconds to a few minutes. Paired-pulse facilitation (PPF) is one of the typical forms of short-term plasticity. STP participates in various neural activities, such as short-term memory and real-time information processing. LTP is a persistent modification of synaptic strength, which can last from hours to years. Long-term plasticity includes Long-term potentiation (LTPo) and long-term depression (LTD). LTPo stimulates the learning of new things, LTD removes unnecessary old information, and both maintain a balance between memory and forgetting of information, so it is regarded as the most important biological mechanism basis of cognition and memory [84]. Henry *et al*. proposed the spike-timing-dependent plasticity (STDP) [85], in which the time difference between two pulses also affects the change of synaptic weights. The timing of the pulse signal is considered on the basis of Hebb’s learning rule. STDP is an extension of the Hebb’s learning rule, which is regarded as a fundamental high-level learning rule in the biological brain. Synaptic weight also varies according to frequency of neuronal signals, which is called spike-rate-dependent plasticity (SRDP) [86]. The Bienenstock, Cooper, and Munro (BCM) theory of synaptic plasticity is the typical model of SRDP and is the most accurate synaptic mode [87]. The behavior of synaptic weight varying with stimulus voltage amplitude is called spike-voltage-dependent plasticity (SVDP), whose properties provide an ideal platform for modelling new neural functions. The SVDP properties are analogous to the Matthew effect, which is a mechanism of evolutionary theory of merit selection, that is ‘the stronger the stronger, the weaker the weaker’, and can be used for modelling neural habituation and sensitization functions, as well as for modelling noise filtering functions [37].

## 3. Materials

**Figure 4.**
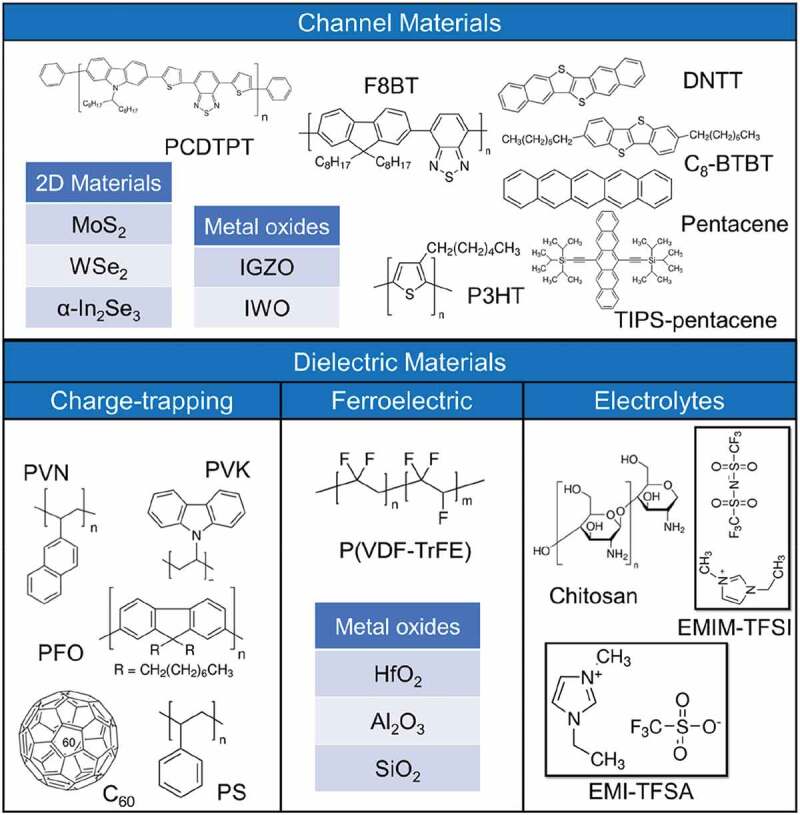
The material used in the fabrication of the pseudo-transistor devices.

Pseudo-transistor is a thin-film system assembled from three essential functional layers: the semiconducting channel, the dielectric, and the electrode materials. As shown in Figure 4, both the chemical and physical properties of channel materials and dielectric materials are critical to the functionality of pseudo-transistors.

### 3.1. Channel materials

In pseudo-transistor devices, the conducting channel is formed in the first few molecular layers of semiconductors near the dielectric layer. Semiconductor materials can be divided into three main types based on the type of majority carriers: (1) *p*-type materials with holes as majority carriers, (2) *n*-type materials with electrons as majority carriers, (3) ambipolar materials.

Owing to the high carrier density and mobility, two-dimensional (2D) channel materials have been widely used to fabricate pseudo-transistor devices [88–91], such as transition metal dichalcogenides (TMDCs, e.g, MoS_2_, WSe_2_). The absence of suspended bonds on the surface of 2D semiconductor materials allow the design of various heterogeneous structures by building ‘Lego’ structures without considering whether the lattice is matched or not [92]. The atomic film thickness is helpful to reduce the power consumption of the device [92,93]. 2D channel materials exhibit excellent compatibility in the adjustment of electronic characteristics [94]. For example, the single-layer MoS_2_ has a direct bandgap of 1.8 eV, Which will transform into an indirect bandgap of 1.4 eV with the increases of MoS_2_ layers, thickness [95]. In addition, the excellent air stable makes TMDs a potential contender for pseudo-transistor channel materials. To further expand the 2D material library, 2D ferroelectric channel materials have been explored [96], such as α-In_2_Se_3_ [97], CuInP_2_S_6_ [98] and BA_2_PbCl_4_. Particularly, α-In_2_Se_3_ can exhibit stable and strong out-of-plane (OOP) and in-plane (IP) ferroelectricity polarization at room temperature [97,99–101], even when the film is monolayer thickness [97,102]. The main methods for the preparation of 2D material films are mechanical exfoliation, epitaxial growth, chemical vapor deposition (CVD) growth and hydrothermal growth [93], of which, mechanical exfoliation method and CVD growth are the common methods for 2D material thin films preparation. The mechanical exfoliation process can only produce small-scale 2D channel materials with uncontrollable and unrepeatable thicknesses. Although the CVD method can solve the problem of large-area film preparation, still a great challenge to control the nucleation process of, resulting in poor film uniformity [103,104].

Organic channel materials can be fabricated over a large area. According to chemical and physical properties, organic semiconductors can be divided into two molecules materials (C_8_-BTBT, pentacene, TIPS-pentacene and DNTT). The crystallinity of small molecule semiconductors is generally better than that of polymers, and higher crystallinity facilitates charge transfer. Compared with *n*-type organic semiconductors, *p*-type organic semiconductors have the following advantages: (1) elevated highest occupied molecular orbital (HOMO), which is conducive to forming good ohmic contact with electrodes and lower the hole injection barrier [105]. (2) Stable chemical properties, which can maintain excellent environmental stability and working stability. (3) Higher carrier mobility.

### 3.2. Dielectric materials

Dielectric materials play an important role in changing the channel conductance. According to working mechanism, dielectric materials can be divided into three categories: (1) charge-trapping materials. Under the premise of matching the energy barrier of the channel material, the charge-trapping material should have a wide band gap to prevent spontaneous charge de-trapping behavior. The most common classes of organic charge-trapping materials are polymer electret, such as poly(2-vinylnaphthalene) (PVN) [77], poly(9,9-dioctylfluorene) (PFO) [106], poly(N-vinylcarbazole) (PVK) [34], polystyrene (PS) [107], *etc*. Nanostructured materials are inside a polymer electret or sandwiched between different dielectrics to act as discrete charge storage centers. Nanostructured materials generally include metal/inorganic nanoparticles (NPs, e.g. Au NPs [108,109], CdSe quantum dots (QDs) [110]), C_60_ [111], etc. Metal oxide materials also have the ability to trap charge (e.g. Nb_2_O_5_) [76]. (2) Ferroelectric materials. Ferroelectric material is a dielectric material with large application potential because its polarization intensity can be precisely regulated by drain. Take P(VDF-TrFE) as an example [112]. The ferroelectric properties of P(VDF-TrFE) are comparable to those of chalcogenide oxide ferroelectric materials. It is widely used in synaptic devices because of its ability to flip dipoles at low voltages. (3) Electrolytes. Electrolytes are ionic conductors, electronic insulators [113]. In memflashes and memtransistors, the electrolyte material can act as a high-capacitance dielectric layer. By applying a drain voltage, ions migrate to form the electrical double layer (EDL) or produce redox reactions [114,115], thus enhancing or depleting the channel carriers. The working mechanism has the advantages of low voltage operation and high linearity of weight update. Electrolyte materials include: Nafion [116], biocompatible materials (e.g. albumin [115], chitosan [117]), ion-gel (e.g. polymeric insulator poly(vinylidene fluoride-co-hexafluoropropylene) (PVDF-HFP) mixed with common ionic liquids, e.g. 1-ethyl-3-methylimidazolium bis(trifluoromethylsulfonyl)imide (EMIM-TFSI) [71], 1-ethyl-3-methylimidazolium trifluoromethanesulfonate (EMI-TFSA) [118]), and PEO-EV(ClO_4_)_2_ [114].

When a dielectric is used in the tunneling/blocker layers, it prevents charges from leaking. There are two main types of tunneling/blocker layer materials commonly applied: (1) 2D materials, such as *h-BN* [119,120]. *h*-BN not only has a large band gap (5.97 eV), but also the clean surface reduces the influence of defects [121]. (2) metal oxides, such as Al_2_O_3_, SiO_2_ and HfO_2_ [122], are used due to their high-*k* insulating properties and stable chemical and physical properties. Meanwhile, the introduction of high-*k* materials can effectively reduce the operating voltage of pseudo-transistors [122].

## 4. Mechanisms, architectures and neuromorphic computing

Artificial neural network (ANN) is the most used software algorithm when it comes to hardware simulation. The dynamic range (DR) and nonlinearity and the LTP/LTP weight update are important metrics of synaptic devices for online learning and offline classification. DR is an index describing the relative range of conductance change, defined as the ratio of *G*_max_ to *G*_min_. The switching ratio determines the dynamic range of the device weight update. The larger the weight range, the more accurate the calculation [123]. In synaptic devices, nonlinearity needs to be introduced to describe the change of conductance with the number of voltage pulses. In neuromorphic computing, the higher the linearity of LTP/LTD, the more accurate the computing results [38].

### 4.1. Tunneling random access memory

**Figure 5.**
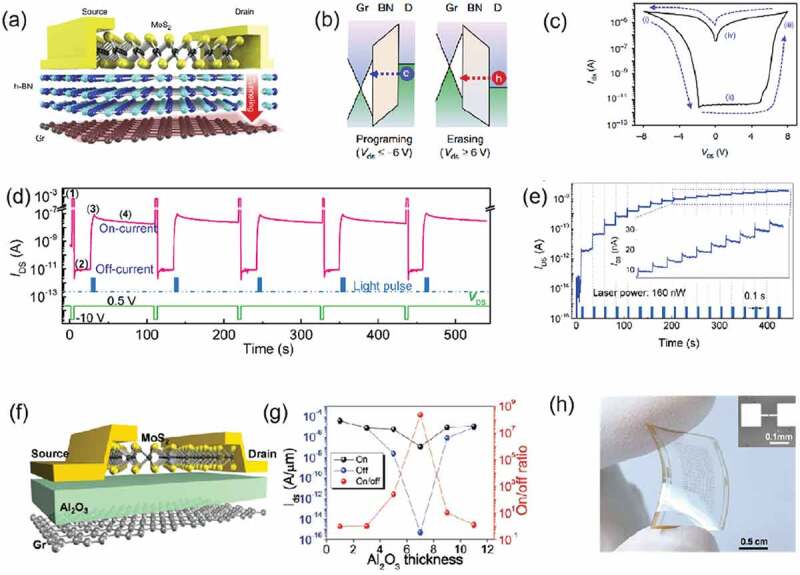
(a) Schematic of the two-terminal TRAM with monolayer MoS_2_ as a semiconducting channel at the top, *h*-BN as a tunneling insulator in the middle and monolayer graphene as a floating gate, charge tunneling between drain and graphene is shown by red arrow. (b) Band diagrams of drain/*h*-BN/graphene, the dashed line arrows indicate the tunneling direction of electrons and holes. Electrons are tunnelling from the drain to graphene at the *V*_DS_ ≤ −6 V and holes are tunnelling from *h*-BN to graphene at *V*_DS_≥6 V states. (c) Typical *I-V* curve of the TRAM with 5.5 nm thick *h*-BN. The current sweep by sweeping *V*_DS_ is shown as a dashed line. The current sweep can be separated into four stages: (i) program, (ii) read, (iii) erase and (iv) read. Channel length and channel width of the device are 4 and 2 mm. Reproduced with permission [65] Copyright 2016 Springer Nature. (d) Operating sequence of the device for five cycles. Each cycle includes: (1) programming by a *V*_DS_-pro pulse (−10 V, 1 s), (2) off-current reading for 20 s, (3) erasing by a 458 nm laser pulse (160 nW, 1 s), and 4) on-current reading for 80 s. The *I*_DS_ was read at *V*_DS_ = 0.5 V. (e) *I*_DS_ curve under with light pulses (*P* = 160 nW, *λ* = 458 nm) every 25 s, exhibiting the capability of multilevel memory. Reproduced with permission [64] Copyright 2019 WILEY-VCH. (f) Schematic of the TRAM device: Graphene floating-gate, MoS_2_ semiconducting channel, and Al_2_O_3_ tunneling insulator. The red arrow indicates charge tunneling between the drain and graphene. (g) On-current (black dots), off-current (blue dots), and on/off ratio (red dots) of TRAM as a function of Al_2_O_3_ thickness. (h) Optical image of TRAM array on the PET substrate. Reproduced with permission [63] Copyright 2017 WILEY-VCH.

The structure of TRAMs is similar to that of traditional floating-gated memory devices, including semiconductor layer, tunneling layer, floating-gate layer, but only source and drain terminals. As for TRAM, during the drain programming process, electrical charge can be injected into the floating gate by tunnelling. Due to the existence of a robust tunneling layer and a charge blocking layer, the trapped charge can be stored nonvoluntarily. Figure 5(a) shows the structure of the TRAM device reported by Lee *et al*. with MoS_2_/*h*-BN/monolayer graphene (Gr) [65]. We can understand its structure as a simplification of the floating-gated transistor structure. The resistance state transition of TRAMs is caused by the source/floating-gate and drain/floating-gate barrier differences (Figure 5(b)). When−6 V is applied to the drain, under the action of the electric field formed by the drain and Gr, the electrons pass through *h*-BN into the Gr layer, and the device changes from LRS to HRS. Due to the good conductivity of Gr, the tunneling electrons will be distributed in the whole floating-gate layer. However, the potential difference between the source and Gr is not enough to drive the tunneling of electrons at the source. However, when+6 V is applied, the conductance changes from HRS to LRS due to the tunneling of holes (Figure 5(c)). Photosensitive semiconductor channel can support an optical terminal to modulate the storage characteristic of TRAM devices. A single layer of MoS_2_ can act as both a conductive channel and an light absorbing layer [64]. When *V*_DS_ = −10 V, the electrons enter the floating-gate layer. Then under the 458 nm light irradiation, the photogenerated holes break through the potential barrier and combine with the electrons to serve as the programming and erasing processes, respectively (Figure 5(d)). Under continuous light pulses (*P* = 160 nW, *λ* = 458 nm), 18 memory states can be obtained (Figure 5(e)). By using optical pulses of different wavelengths, it is proved that the photogenerated holes are generated in MoS_2_ rather than excited by *h*-BN. Logic circuits such as ‘AND’ and ‘OR’ gates can be simply implemented by combining optical inputs with electrically driven MoS_2_/*h*-BN/graphene heterostructures. However, the thickness of the tunneling layer also affects the threshold voltage and switching ratio. Yu *et al*. replaced *h*-BN with Al_2_O_3_ (Figure 5(f)), and the thickness of Al_2_O_3_ could be easily changed using atomic layer deposition (ALD) process. With the thickening of Al_2_O_3_ film from 1 to 7 nm, the switching ratio of the device increases due to the increased barrier between the source and the floating gate. Al_2_O_3_ thicknesses greater than 7 nm can cause the device off-state current to be higher (Figure 5(g)). Likewise, the tunneling voltage threshold will increase as the tunneling layer thickness increases [63]. In recent report, flexible TRAM has been reported by using MoS_2_ as channel material and Al_2_O_3_ as tunneling layer [63], and the bending strain of the device can reach 1% (Figure 5(h)).

### 4.2. Memflash

**Figure 6.**
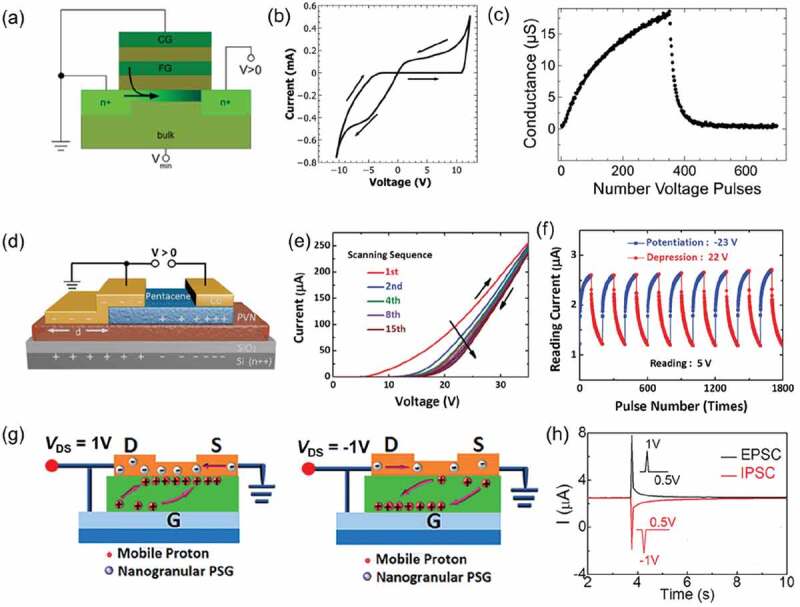
(a) in GS model, at positive voltages, applied to the drain terminal (*V* = 0 V), electrons are tunneling from the floating-gate through the gate oxide layer to the conducting channel of the underlying MOS transistor. (b) at negative voltages (*V* < 0 V) the electrons are recaptured. Reproduced [66] Copyright 2012 AIP Publishing. (c) Appropriate voltage pulses can be used to mimic the process of synaptic depression. A train of potentiation voltage pulses (10 V, 0.1 s) are applied, with intermittent reading at 2.5 V. A train of depression voltage pulses (−7 V, 0.2 s) are applied, with intermittent reading at 2.5 V. Reproduced [67] Copyright 2013 AIP Publishing. (d) Device structure of an organic memflash, in which accumulated charge distribution under positive bias is schematically illustrated, two Cu top electrodes are asymmetric in size, and d denotes the difference in the electrode length. (e) Under consecutive positive voltage sweeps (from 0 V to 35 V, then back to 0 V), showing continuous decrease in current. (f) Synapse-like potentiation and depression. Depression: a train of programming voltage pulses (22 V, 0.2 s) are applied, with intermittent reading at 5 V. Potentiation: a train of erasing voltage pulses (−23 V, 0.2 s) are applied, with intermittent reading at 5 V. Reproduced [125] Copyright 2018 WILEY-VCH. (g) Schematic image of the MPEC hybrid IWO transistor working in the GD model. (h) EPSC/IPSC triggered on the MPEC hybrid IWO transistor working in GS mode. Reproduced with permission [124] Copyright 2017 AIP Publishing.

A single floating gate transistor with memristor operating mode is proposed by Ziegler *et al* [66], namely memflash. The device structure of memflash is divided into two wiring modes: (1) GS mode: the gate is shorted to the source. (2) GD mode: the gate is shorted to the drain. In a typical memflash device, when a voltage is applied to the drain, an *I-V* hysteresis curve can be observed. It is reported that the memflash mechanism relies on electron tunneling (floating-gate charge/discharge process) or ion migration (formation of EDL) [124]. No matter what wiring method is used, precise tuning of the drain conductance can be achieved, but it is still essentially a floating-gate transistor for electrodes are connected to each other, they have the same potential. In GS mode, the gate voltage is equal to the source voltage, the conductance variation is completely controlled by the drain, and the gate does not affect the carrier variation in the channel. Compared to GS mode, in GD mode, the gate voltage is equal to the drain voltage and is not 0 V, which leads to the gate also playing the role of regulating the channel current.In GS mode, *n*-channel memflash based on the electron tunneling mechanism is discharged (Figure 6(a)) or charged at the floating-gate in the case where the drain is applied with positive voltage. Figure 6(b) shows its resistance transition process, embodied in: (1) under the positive drain voltage, electron tunneling through the gate oxide from the floating-gate layer to the conductive channel, resulting in the transition of HRS to LRS. (2) When negative voltage is applied, the charge in the conductive channel is captured by the floating-gate layer again, and the conductance state changes from LRS to HRS. Ziegler *et al*. verified the ability of memflash to mimic synaptic plasticity [67]. As shown in Figure 6(c), the as fabricated memflash demonstrated LTP/LTD updating after a series of pulsed stimuli. *P*-channel memflash exhibits the opposite resistive switching [126]. By combining the two types (*n* and *p*-channel) of memflash devices into a single two-terminal device structure, complementary resistive switching device with bipolar symmetric switching characteristics can be obtained and rich logic functions can be realized. However, memflash has a longer programming time (in the order of milliseconds) and higher programming voltage due to the relatively thick tunnel layer. The thickness of the tunneling layer is determined by a trade-off between long-term memory properties and short-term synaptic plasticity, and lower operating voltage and power consumption are achieved by reducing the thickness of the oxide layer [68]. In addition, the reduced thickness of the oxide layer improved the linearity of the weight update [68]. Zhong *et al*. studied the electrode topology transformation of floating-gated transistor memory [125]. Because the gate and drain of the transistor are grounded and there is not a charge exchange between the equipotential bodies, the two electrodes (gate and source) can be combined to form a large electrode, that is, the source and drain electrode form an asymmetric structure (Figure 6(d)). It is worth noting that the silicon substrate is completely floating and acts as a floating-gate. The surface charge density of the drain, channel and source decreases in turn, and the resulting vertical electric field also decreases sequentially. Since charge trapping requires sufficient electric field to induce carriers injection into the dielectric layer, the charge trapping/de-trapping effect will only occur on the drain side. In addition, the charge trapping material should have the ability to maintain long-term storage of charge, and the device can be read stably at each conductance state. During the process of hole trapping, the resistance slowly changes from LRS to HRS (Figure 6(e)). While during the de-trapping process of holes, the resistance changes slowly from HRS to LRS in succession. The consecutive change of resistance states and the non-volatile memory property provide the possibility for the simulation of synaptic plasticity behavior. As shown in Figure 6(f), the asymmetric electrode-based memflash shows a continuous increase or decrease in *I*_DS_ under *V*_DS_ stimulation, a phenomenon that well mimics the synaptic continuous LTP/LTD transition. Recently, Gong *et al*. reported a memflash device with CsPbBr_3_ quantum dots [127]. With the charge trapping/de-trapping of CsPbBr_3_ quantum dots/Al_2_O_3_ junction, the current changes shown in LTP and LTD have high reconfigurability and a large range of weight update, which can effectively sense weak ultraviolet light. Zhu *et al*. reported a memflash device of mixed protonic and electronic conductor (MPEC) hybrid indium-tungsten-oxide (IWO) [124]. Synaptic functions have also been mimicked on solid-state MIEC hybrid IWO memflash, such as excitatory post-synaptic current (EPSC) and inhibitor post-synaptic current (IPSC). Figure 6(g) shows the working mechanism of MPEC hybrid IWO memflash in GD mode. With working in the GD mode, the MPEC hybrid IWO memflash could be used to mimic the postsynaptic currents (Figure 6(h)). The migration of protons in an electrolyte to the electrolyte/semiconductor interface (or electrolyte/gate interface) under a positive(or negative) stimulating voltage. However, in GS mode, the positive stimulation voltage causes proton migration towards the electrolyte/gate interface, resulting in the reverse phenomenon.

### 4.3. Memtransistor

**Figure 7.**
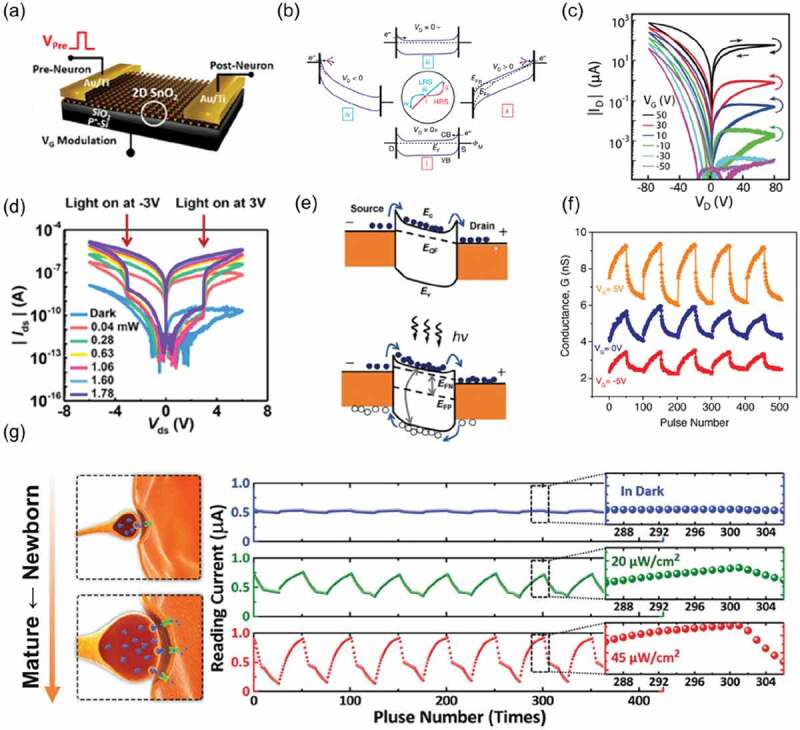
(a) Schematic of the inverted-staggered FET structure using the 2D-SnO_2_ memristor channel. Reproduced with permission [128] Copyright 2021 American Chemical Society. (b) Schematic showing the energy band diagram of an LRS-LRS memtransistor at the four switching stages shown in the center, *E*_Fp_ and *E*_Fn_ are non-equilibrium quasi-Fermi levels for holes and electrons, C_B_ and V_B_ stand for conduction band and valence band, and purple arrows show defect migration. (c) *I*_D_*-V*_D_ curves for ten consecutive sweeps at each gate bias *V*_GS_ for the same device. The switching directions are shown by the curved arrows. *V*_GS_ was decreased for each consecutive sweep cycle. Reproduced with permission [69]. Copyright 2018 Springer Nature. (d) a three-layer MoS_2_ memtransistor device capable of electrical, optical and combined modulation, *I*_DS_-*V*_DS_ curves in the dark and under modulation of different light powers (0.04, 0.28, 0.63, 1.06, 1.60, and 1.78 mW). *V*_GS_ is −80 V during the measurement, and light was on once *V*_DS_ reaches 3 or −3 V. (e) in *V*_GS_ = 0 V and under illumination, the band diagram of Au/MoS_2_/Au memtransistor. The quasi-Fermi level (*E*_QF_) formed under electric field is displayed as dashed line. The full dots at the conduction band energy level (*E*_c_) and the empty dots at valence band energy level (*E*_v_) in MoS_2_ denote the electrons and holes, respectively. The splitting of the Fermi level under illumination is highlighted as a quasi-Fermi level of electrons (*E*_FN_) and quasi-Fermi level of holes (*E*_FP_). Reproduced with permission [94] Copyright 2019 American Chemical Society. (f) in the inverted-staggered FET, repeatable potentiation and depression behaviors with different gate voltages. Reproduced with permission [128] Copyright 2021 American Chemical Society. (g) Schematics of organic memtransistors operating in dark, and under relatively weak (20 μW cm^−2^) and strong (45 μW cm^−2^) UV illumination, mimicking different synapses from newborn to mature stages. For synaptic depression, a train of programming pulses (10 V, 0.2 s) with intermittent reading (5 V, 0.2 s) are applied. For synaptic potentiation, a train of erasing pulses (−10 V, 0.8 s) with the same reading are applied. Reproduced with permission [77] Copyright 2019 WILEY-VCH.

A memtransistor can be thought of as a memristor controlled by the gate voltage with the drain electrode as the main drive terminal (Figure 7(a)) [9,122,128]. Compared with memflash and TRAM, memtransistor can not only precisely control the resistance switching behavior between source and drain by constant gate voltage, but also can achieve competitive regulation by simultaneously applying input signals from drain and gate [91]. This property makes memtransistors as non-volatile memories with great potential in mimicking synaptic plasticity, especially in heterogeneous synaptic plasticity. Sangwan *et al*. reported the first memtransistor using polycrystalline monolayer MoS_2_ films as the channel material, demonstrating that the transition in channel conductance results from dynamic changes in the Schottky barrier (Figure 7(b)) [69]. Changing the gate voltage not only modulates the switching ratio of hysteresis window, but also changes its transition behavior of resistance state (Figure 7(c)). For example, the non-volatile resistance state is converted from HRS to LRS at high gate voltage and from HRS to LRS at low gate voltage. Yin *et al*. used a light gate to modulate the memtransistor hysteresis curve (Figure 7(d)) [94]. The surface state of MoS_2_/Au interface can trap photogenerated carriers under light, reducing the height of the potential barrier (Figure 7(e)). The experimental results indicated that grating modulation increases the hysteresis switching ratio by two orders of magnitude compared to that of electric gate modulation. A higher hysteresis switching ratio could be achieved based on optoelectronic co-regulation. Recent study has shown that the photoelectric properties of memtransistors can be further improved by using heterojunctions. Nguyen *et al*. reported a memtransistor device with a switching ratio of up to 10^6^ at operating voltages (<10 V) using a two-dimensional Te/ReS_2_ van der Waals heterostructure encapsulated by Al_2_O_3_ [129].

It is worth noting that the modulation of resistance switching characteristics could also be achieved by adjusting ion migration and charge trapping in memtransistors. Zheng *et al*. prepared an organic memtransistor based on charge trapping mechanism [130]. In the self-assembly process, hydrogen bond aggregation between (3-Aminopropyl) triethoxysilane (APTES) molecules will form defects on the interface to realize the trapping/de-trapping effect. It can not only affect the charge density in the channel through the field effect through the voltage polarity of the gate, but also adjust the intensity of the external electric field through the cooperation or competition between the gate and the drain to change the capture ability. In the latest report, Das *et al*. reported a memtransistor using conjugated polymer thin-film and redox-active solid electrolyte [114]. It is based on the redox reaction between the dissociated ethyl viologen (EV^2+^) ions in the dielectric layer and the P3HT monomer unit of the polymerization backbone in the active channel, which realizes typical synaptic functions such as EPSC, PPF, LTP and STDP. In addition, it features low operating voltage (<3 V) and low power consumption (<250 pJ).

Synaptic plasticity can be regulated by both the drain and the gate of the memtransistor. Memtransistors can simulate heterogeneous synaptic plasticity for precise tuning of the dynamic range without any additional tuning terminals and external devices (Figure 7(f)) [128]. The random variation of quantitative defect state between devices is likely to lead to unstable updating of synaptic weights. Therefore, it is essential to find a way to induce and achieve stable conductance changes. The insertion of Nb_2_O_5_ between the MoS_2_ channel and the gate insulation layer to increase the barrier height, and increase the number of different conductivity states (>200). This will further improve the linearity of the weight update and achieve a large range of tunability of the weight update [76]. Zhong *et al*. reported a photosensitive memtransistor using pentacene as the channel material, PVN as charge trapping material [77]. The rate of hole in and out between the pentacene and PVN was determined by the intensity of ultraviolet light, and the hysteresis switching ratio of the device was controlled by changing the light intensity. The change of light intensity changes the weight updating range, which is similar to the developmental behavior of synapses. In addition, we note that the joint action of gate and drain to regulate the capture capability of transistor also provides a new way for memory development.

Memtransistor devices feature gate-regulated source-drain conductance state and their LTP/LTD linearity can be optimized by the gate inputs. Cho *et al*. reported a MoS_2_/ZrO_2-x_ heterostructure memtransistor [131]. The nonlinearity of MoS_2_/ZrO_2-x_ memtransistor conductance for drain or gate pulses is 0.52 and 1.72, and the maximum identification accuracy is 90% and 87%, respectively. The conductance nonlinearity is−0.91 with a recognition rate up to 92% under the joint action of the gate and drain.

Memtransistors can be used to implement higher-order spatio-temporal information processing. The Bienenstock, Cooper, and Munro (BCM) theory of synaptic plasticity is a typical model of SRDP. The sliding frequency threshold and the enhanced depression effect (EDE) are two of the most critical features of the BCM rule. In spite of the fact that the frequency-based BCM rule has been proved to be achievable using two-terminal memristors, there is no EDE during the process of weight updating. On the contrary, the gate of memtransistor provides an additional competing factor. For example, Han *et al*. reported a memtransistor based on MoS_2_/WSe_2_ heterojunction [132]. The heterojunction structure can effectively reduce the defect energy level in the two-dimensional material and realize the spontaneous forgetting process. At the same time, the gate can regulate the mobility of channel carriers through field effects, which in turn regulates the forgetting rate, and the gate modulation effect of memtransistor promotes the realization of EDE.

## 5. Conclusions and perspective

In summary, we have reviewed the recent progress of pseudo-transistor based neuromorphic devices. A summary of device metrics for demonstrated pseudo-transistor devices are provided in Table 1. At present, pseudo-transistor is in the preliminary stage of development and exploration, and there are still a number of problems and challenges need to be solved.

1. Materials: For 2D materials, the optimization of the film preparation process to achieve large-area preparation of films and improve the uniformity of the film are crucial problems. The main challenge for the future development of organic pseudo-transistors is the synthesis of new organic semiconductor materials with high mobility, solution processability and air stability. By utilizing photosensitive materials, light is taken as one of the dimensions of pseudo-transistor performance regulation to achieve bionic simulation of human eye vision, and electrolyte materials are used to simulate skin sensation to different humidity environments.
2. Performance: For large scale integrated array, power consumption is one of the most important technical indicators of synaptic devices. Besides, large operating voltage will result in slow operating speed. For TRAM, charge injection past the tunneling dielectric is typically governed by Fowler-Nordheim tunnelling, therefore, requires large voltages (>8 V). Although MPEC hybrid IWO memflash show a low-voltage operation mechanism, the signal pulse width is large (>120 ms) depending on the nature of the electrolyte. Beyond that, a linear conductance-update process is another functional parameter in neuromorphic computing. Although, the gate of memtransistor has an adjusting effect on LTP/LTD linearity, the gate increases the complexity of the array. For TRAM and memflash, we also need to modulate the relaxation timescale required from synaptic plasticity to non-volatile storage to further improve the accuracy of neuromorphic computations. For example, the oxide thickness is reduced to improve the linearity of LTP/LTD, but at the expense of its original maintenance performance.
3. Function: In neuromorphic computing, the ultimate goal is that the device can stably realize the fusion of ‘perception, memory, and computation’. Pseudo-transistors are still in the early stages of simulating synaptic function. To achieve more complex neuromorphic functions (e.g. neuron, dendrites and soma), it is necessary to introduce multi-physics mechanisms or make topological changes in the structure based on the existing studies of pseudo-transistors.
4. Circuit integration: The achievement of a reproducible pseudo-transistors with a high device yield has become a significant challenge in the development of neuromorphic circuits. Generally, the unavoidable temporal (cycle-to-cycle) and spatial (device-to-device) variations are originated from the stability of the pseudo-transistor and the inter-device uniformity in the large-scale preparation process. Accurately controlling the homogeneous growth of each functional layer in the large-scale preparation process, puts higher requirements on the preparation process.

**Table 1.** Summary of Pseudo-Transistors research progress.

Device Type	Channel material	Dielectric material	Mechanism	Switching ratio	Retention [s]	Endurance [cycles]	Operating Voltage [V]	Conductance number (#)	Ref.
TRAM	MoS_2_	*h*-BN	Tunnel	~10^9^	10000	10^5^	8	–	[65]
	MoS_2_	Al_2_O_3_	Tunnel	~10^3^	1000	8000	8	6	[63]
	MoS_2_	*h*-BN	Tunnel	~10^6^	10000	10^4^	12	18	[64]
Memflash	IWO	PSG	Ion migration	–	–	–	1.5	–	[124]
	Pentacene	PVN/SiO_2_	Charge trapping	~10^5^	1000	–	23	5	[125]
	Pentacene	Al_2_O_3_/CspbBr_3_/SiO_2_	Charge trapping	–	–	–	50	–	[127]
Memtransistor	MoS_2_	SiO_2_	Defect-induced Schottky barrier change	~100	90000	500	40	11	[69]
	IGZO	lon-gel/Al_2_O_3_	Trap charge electric-double-layer	~10^6^	–	–	4	30	[71]
	GaSe	SiO_2_	the formation and rupture of the metallic filament	~10^5^	10^4^	5000	20	–	[72]
	MoS_2_	SiO_2_	Charge trapping	~10^3^	10^5^	250	20	10	[91]
	*h*-BN/graphene/MoS_2_	SiO_2_	the formation and rupture of the metallic filament	~10^6^	–	40	6	2	[120]
	Pentacene	APTES/SiO_2_	Charge trapping	~10^2^	10^4^	100	50	100	[130]
	MoS_2_	HfO_x_	Defect-induced Schottky barrier change	~10^4^	–	160	12	250	[122]
	SnO_2_	SiO_2_	Defect-induced Schottky barrier change	~10	10^4^	1000	20	50	[128]
	MoS_2_	SiO_2_	Schottky barrier change	~10^5^	–	–	8	–	[94]
	Te/ReS_2_	Al_2_O_3_/SiO_2_	Defect-induced Schottky barrier change	~10^6^	13000	200	8	16	[129]
	MoS_2_	Nb_2_O_5_/SiO_2_	Charge trapping	~10	1000	200	14	50	[76]
	Pentacene	PVN/SiO_2_	Charge trapping	~10^3^	1000	–	10	25	[77]
	VO_x_	VSiO_x_/SiO_2_	Charge trapping	~6	–	6000	1	20	[133]
